# Lactate alleviates intestinal barrier injury in weaned piglets via activation of the Wnt/β-catenin pathway and promotion of intestinal epithelial cell proliferation

**DOI:** 10.1186/s40104-025-01290-x

**Published:** 2025-11-28

**Authors:** Mingyu Wang, Yifan Chen, Jiaojiao Chen, Aimin Wu, Daiwen Chen, Bing Yu, Jun He, Jie Yu, Xiangbing Mao, Zhiqing Huang, Yuheng Luo, Junqiu Luo, Ping Zheng

**Affiliations:** 1 Institute of Animal Nutrition, Sichuan Agricultural University, Key Laboratory of Animal Disease-Resistance Nutrition, Ministry of Education, and Key Laboratory of Animal Disease-Resistant Nutrition, Sichuan Province, Chengdu, 611130 People’s Republic of China; 2 College of Animal Science and Technology, Hebei Agricultural University, Hebei, 071000 People’s Republic of China

**Keywords:** Apoptosis, Intestinal inflammation, Lactate, Lgr5, Piglets, Proliferation, Wnt/β-catenin pathway

## Abstract

**Background:**

Inflammatory bowel disease causes intestinal structural damage, impairs gut function, hinders animal growth and development, and reduces farming efficiency. Previous studies demonstrated that lactate alleviates dextran sulfate sodium (DSS)-induced inflammation and mitigates weight loss by enhancing intestinal barrier functions. However, ‌the mechanisms underlying‌ lactate-mediated protection of the intestinal epithelial barrier ‌remain unclear‌. This study aimed to explore the protective effect of lactate on intestinal barrier damage in colitis piglets and the possible underlying mechanisms through in vivo and in vitro experiments.

**Methods:**

A total of 60 21-day-old weaned female piglets were randomly assigned into three groups based on weight: the control group (basal diet with physiological saline gavage), the DSS group (basal diet with 5% DSS gavage), and the DSS + LA group (2% lactate diet with 5% DSS gavage). There were 10 replicates per treatment, with 2 piglets per replicate. Jejunal morphology was assessed via hematoxylin and eosin staining, while Western blotting quantified the protein levels of proliferation markers, including cluster of differentiation 24 (CD24), cyclin D1, and wingless/integrated (Wnt)/β-catenin signaling components. In vitro, 0.08% DSS and 2–32 mmol/L sodium lactate-treated intestinal porcine epithelial cell line-J2 (IPEC-J2) cells (*n* = 4) were assessed for viability (Cell Counting Kit-8 assay), apoptosis (flow cytometry), and proliferation parameters, including cell cycle analysis and Leucine-rich repeat-containing G-protein coupled receptor 5 (Lgr5^+^) stem cell quantification.

**Results:**

In vivo, DSS administration induced jejunal villus shortening (*P* < 0.05), downregulated protein levels of CD24, cyclin D1, casein kinase 1 (CK1), and dishevelled-2 (DVL2) (*P* < 0.05). In vitro, DSS promoted apoptosis, inhibited proliferation, diminished the Lgr5^+^ cell populations (*P* < 0.05), and reduced S-phase cell proportions (*P* < 0.05). Conversely, lactate supplementation ameliorated DSS-induced villus atrophy (*P* < 0.05), restored CD24, cyclin D1, CK1, and DVL2 protein levels (*P* < 0.05). Furthermore, in vitro, sodium lactate attenuated DSS-induced apoptosis (*P* < 0.05), enhanced IPEC-J2 proliferation (*P* < 0.05), expanded Lgr5^+^ cells (*P* < 0.05), and increased S-phase progression (*P* < 0.05).

**Conclusions:**

In summary, lactate ameliorated intestinal barrier damage in DSS-induced colitis by activating the Wnt/β-catenin pathway and restoring the balance between epithelial cell proliferation and apoptosis. This study provides novel mechanistic evidence supporting lactate’s therapeutic potential for IBD management.

**Supplementary Information:**

The online version contains supplementary material available at 10.1186/s40104-025-01290-x.

## Introduction

Inflammatory bowel disease (IBD), while originally characterized in humans, describes chronic intestinal inflammatory conditions that also pose significant threats to animal health and the livestock industry. Studies have shown that IBD disrupts intestinal barrier function by disturbing the dynamic balance between cell proliferation and apoptosis [1, 2], leading to increased intestinal permeability. This allows endotoxins and antigens from the gut to enter the bloodstream, triggering systemic inflammatory responses such as sepsis [3]. Impaired intestinal barrier function in animal production results in reduced growth performance, including weight loss [4], decreased average daily gain [5], and lower feed conversion efficiency [6]. In severe cases, it can threaten animal survival and reduce farming profitability. The dextran sodium sulfate (DSS)-induced colitis model has become instrumental in IBD research, reliably recapitulating key features including mucosal ulceration, epithelial apoptosis/proliferation imbalance, and barrier dysfunction [7–11].

A key factor in IBD pathogenesis is the imbalance between apoptosis and cell proliferation. Excessive immune activation accelerates epithelial apoptosis [12–14], initiating a self-perpetuating cycle of "inflammation–apoptosis–increased permeability", leading to epithelial shedding, increased luminal antigen permeability, and the release of various damage-associated molecular patterns (DAMPs) [15]. Intestinal stem cells (ISCs), particularly the Lgr5⁺ subpopulation, undergo rapid proliferation under the regulation of the Wnt/β-catenin signaling pathway during inflammation, thereby compensating for the loss of intestinal epithelial cells and driving mucosal repair [16–18]. In a murine model of sepsis-induced intestinal barrier injury, induction of Lgr5⁺ expression in the colon accelerated epithelial cell proliferation while suppressing apoptosis, thereby preserving colonic morphology under TNF-α–induced damage [19]. Conversely, depletion of the ISC pool during colitis impairs epithelial self-renewal, further exacerbating barrier defects and amplifying inflammation [20, 21]. Previous studies have demonstrated that STAT6-dependent M2 macrophages secrete Wnt ligands to activate Wnt/β-catenin pathway, driving ISC-mediated mucosal repair [22]. The dual role of Wnt/β-catenin is noteworthy: (i) promoting the rapid proliferation of Lgr5⁺ ISCs by regulating cell cycle progression via cyclin D1 [19, 23–25], and (ii) exerting anti-apoptotic effects in extra-intestinal tissues [26, 27]—a mechanism that remains underexplored in porcine inflammatory bowel disease.

Lactate is not only the end product of glycolysis but also functions as a critical signaling molecule and metabolic substrate essential for maintaining immune homeostasis. In a porcine model of fecal peritonitis, piglets with reduced circulating lactate levels exhibited significantly higher mortality, underscoring the pivotal role of lactate in preserving systemic homeostasis under inflammatory conditions [28]. A previous study analyzed the correlation between serum biomarkers of intestinal permeability (zonulin and intestinal fatty acid-binding protein, I-FABP) and tissue levels of lactate or the lactate-to-pyruvate ratio [29]. They found that lower lactate concentrations or lactate/pyruvate ratios in intestinal tissues were associated with increased permeability and impaired barrier function, highlighting lactate as a critical factor of barrier integrity [29]. Consistently, studies in mammalian models of TNBS-induced [30] and DSS-induced intestinal inflammation [31] have demonstrated that lactate effectively attenuates inflammation-associated disruption of intestinal structure and barrier function. These protective effects are associated with the ability of lactate to stimulate intestinal stem cell proliferation and enhance tissue repair capacity [32, 33]. Previous study revealed that lactate stimulated Lgr5⁺ stem cell expansion by activating the Gpr81 receptor and Wnt/β-catenin signaling, which in turn protected murine intestines against chemo- and radiotherapy-induced injury [33]. Similar effects have been verified in porcine jejunal organoid models [32]. However, the role of lactate in promoting repair via Wnt/β-catenin signaling and ISCs during inflammatory bowel disease in pigs remains insufficiently explored.

Despite established links between lactate and epithelial proliferation, its capacity to rescue Wnt/β-catenin signaling during active inflammation—specifically in modulating both proliferative and anti-apoptotic responses—remains unverified in porcine models. Given our preliminary data showing lactate's mitigation of DSS-induced growth retardation [34], we hypothesize that lactate preserves intestinal integrity during colitis by coordinately enhancing Wnt/β-catenin-driven proliferation while suppressing apoptosis. The jejunum serves as the principal location for nutritional digestion and absorption, and preserving its structural integrity is essential for the growth and development of piglets [35, 36]. Therefore, this study aims to investigate whether lactate can mitigate DSS-induced structural damage in the jejunum by regulating intestinal epithelial cell proliferation and apoptosis. The impact of lactate on intestinal morphology, inflammatory markers, apoptosis, and proliferative pathways was evaluated using both in vivo (weaned piglets) and in vitro (IPEC-J2 cells) models. Our results will offer novel insights into the potential use of lactate as a feed additive to mitigate growth deficits caused by intestinal inflammation through concurrent enhancement of proliferation and inhibition of apoptosis.

## Materials and methods

### Animals and experimental design

This study was approved by the Animal Care Committee of Sichuan Agricultural University (SAU20210160) and carried out following the National Research Council’s Guide for the Care and Use of Laboratory Animals. The experiment used a completely randomized design. A total of 60 healthy female DLY (Duroc × Landrace × Yorkshire) piglets (8.02 ± 0.25 kg) were weaned at 21 days of age. After 3 days of pre-feeding, the piglets were randomly divided into three groups based on body weights: the control group, the DSS group, and the DSS + LA group. The dietary treatments included the addition of 0% (control group and DSS group) or 2% lactate (purity ≥ 80%, JDLAP80, Henan Jindan Lactate Technology Co., Ltd., Dancheng, Henan, China) to a corn- and soybean-based diet. There were 10 replicates per treatment, with two piglets per replicate. Two piglets within the exact replicate were housed in one nursery pen (4.0 m × 3.0 m). Throughout the experiment, piglets had ad libitum access to water and feed. The room temperature was maintained at 28 ± 1 °C. After feeding the experimental diets for 7 d, the DSS and DSS + LA groups were continuously gavaged with a 5% DSS solution for 4 d. On d 8 of the experiment, piglets were gavaged 200 mL of 5% DSS solution, followed by 100 mL of DSS solution on d 9–11. Piglets in the control group were gavaged with an equal volume of physiological saline. The 5% DSS solution was prepared by dissolving DSS powder (YEASEN Biotechnology, Shanghai, China; molecular weight: 36,000–50,000) in physiological saline. Twenty-four hours after the final gavage, one piglet per replicate was euthanized by intramuscular injection of sodium pentobarbital at a dose of 200 mg/kg BW. The jejunal tissue was immediately collected and fixed in 4% neutral formalin solution for subsequent histological analysis. The jejunal mucosa was separated and stored at −80 °C for subsequent Western blot analysis. The remaining piglet in each replicate was continuously fed until the end of the experiment and then subjected to harmless disposal. The diets were formulated according to the nutritional requirements of the National Research Council [37]. Table S1 shows the nutritional composition of the basal diet lactate diet.

### Jejunum morphology

The jejunal tissue samples were fixed with 4% paraformaldehyde and sectioned for histological analysis. Hematoxylin-eosin (H&E) and periodic acid-Schiff (PAS) staining were performed for morphological observation. H&E staining was used to evaluate the overall tissue morphology, including villus and crypt structures, while PAS staining was performed to assess goblet cell abundance and mucus secretion, serving as indicators of jejunal inflammation severity. The villus height, crypt depth, and number of goblet cells were measured. Ten suitable fields of view (with intact and straight villi) were selected from each section for imaging using ImageJ 6.0 software. The images were analyzed, and 30 intact villi were measured for villus length and crypt depth.

### Western blot analysis

Western blot was used to measure the protein levels of proliferating cell nuclear antigen (PCN), cluster of differentiation 24 (CD24), cellular myelocytomatosis virus (c-Myc), cyclin D1, β-catenin, adenomatous polyposis coli (APC), casein kinase 1 (CK-1), dishevelled-2 (DVL2), low density lipoprotein receptor-related protein 5 (LPR5), Frizzled 6, and Axin2 in the jejunal tissues of piglets. Total proteins from the jejunum were extracted using radio-immunoprecipitation assay (RIPA) lysis buffer (Beyotime, Shanghai, China). Intestinal tissue samples (20 µg) were placed into a sterile eppendorf tube, followed by the addition of 400 µL RIPA lysis buffer to each tube. The samples were lysed on ice at 4 °C for 30 min and then centrifuged at 12,000 × *g* for 10 min at 4 °C, and the supernatant was collected. Protein concentration in the intestinal tissues was determined using a BCA protein assay kit (Pierce, Rockford, IL, USA) and a NanoDrop 2000c spectrophotometer (ThermoFisher Scientific, Waltham, MA, USA). The 5 × protein loading buffer (Beyotime, Shanghai, China) was mixed with the protein lysate in a 1:4 ratio and denatured at 98 °C in a metal bath for 10 min. Proteins (10 µg) were separated by gel electrophoresis and transferred to a polyvinylidene fluoride (PVDF) membrane (Millipore, Eschborn, Germany) using a wet Trans-Blot system (Bio-Rad, Hercules, CA, USA). The PVDF membrane was sealed at room temperature with 5% bovine serum albumin (Beyotime) for 2 h, followed by overnight incubation with the primary antibody at 4 °C. The membrane was washed three times with Tris-buffered saline/Tween (TBST), each wash for 10 min, and then incubated with the secondary antibody at room temperature for 1.5 h. Protein signals were captured using BeyoECL Plus (Beyotime) and the ChemiDoc XRS imaging system. Protein levels was analyzed with the Gel-Pro Analyzer and normalized to β-actin protein.

### Culture of IPEC-J2 cells

The porcine intestinal epithelial cell line IPEC-J2 was obtained from the American Type Culture Collection (ATCC). IPEC-J2 cells were cultured in cell culture flasks containing DMEM/F12 complete medium (supplemented with 10% FBS, 100 IU/mL penicillin, 100 mg/mL streptomycin, 5 mg/mL hEGF, and 10 nmol/L HEPES) and incubated at 37 °C with 5% CO_2_ in a humidified incubator. When the cells reached approximately 70% confluence, they were digested with diluted trypsin and collected by centrifugation at 800 ×* g* for 2 min. A new DMEM/F12 complete medium was added to resuspend the cells gently. The cell concentration was then adjusted by counting the viable cells using a cell counter, and cells were seeded onto cell culture plates (96-well plates: 100 µL/well; 12-well plates: 1 mL/well) for subsequent experimental treatments.

### Cell treatments

To determine the effective concentration of DSS for establishing an inflammatory model in IPEC-J2 cells, cells were exposed to different concentrations of DSS (0.01%, 0.04%, 0.08%, 0.1%, and 0.5%) for 24 h. To investigate the role of sodium lactate in alleviating DSS-induced inflammation in IPEC-J2 cells, the following treatments were applied: control (no DSS, 0 mmol/L sodium lactate), DSS only (DSS + 0 mmol/L sodium lactate), and DSS combined with varying concentrations of sodium lactate (2, 4, 8, 16, and 32 mmol/L). To distinguish the effects of lactate anions and hydrogen ions on IPEC-J2 cells, the following groups were designed: control, DSS only, DSS + 8 mmol/L sodium lactate, and DSS + 8 mmol/L HCl. Cells were pretreated with sodium lactate or HCl for 12 h, followed by exposure to DSS for 24 h. Each treatment was performed with four replicates.

### CCK8 cell viability assay

IPEC-J2 cells were seeded into 96-well cell culture plates at a density of 1 × 10^4^ cells/well, with 100 μL of cell suspension per well. Following different treatments, 10 μL of CCK8 solution was added to each well, and the plates were incubated at 37 °C with 5% CO_2_ for 1 h. The absorbance at 450 nm was then measured using a microplate reader (BioTek, Vermont, USA). Cell viability was then calculated according to the instructions provided with the CCK-8 assay kit (Beyotime, Jiangsu, China).

### Real-time quantitative PCR

Gene expression of interleukin 1β (*IL-1β*), *IL-6*, and *IL-18* in IPEC-J2 cells was measured by real-time quantitative polymerase chain reaction (RT-qPCR). Cells at a density of 1 × 10^5^ per well were seeded into 12-well plates and cultured until attachment. Following different treatments, total RNA was extracted from the samples using TRIzol reagent (TaKaRa Biotechnology, Dalian, China), and cDNA was synthesized using the PrimeScripte RT reagent kit (TaKaRa Biotechnology, Dalian, China) according to the manufacturer’s instructions. Relative gene expression was calculated using the ∆∆CT method, and the results were normalized to the *β-actin* gene expression level. The sequences of the gene-specific primers are listed in Table S2.

### Cell apoptosis assay

IPEC-J2 cells were seeded into 12-well cell culture plates at a density of 1 × 10^5^ cells/well, with 1 mL of cell suspension per well. Following different treatments, cells were collected into sterile eppendorf tubes and centrifuged at 600 × *g* for 5 min. The supernatant was discarded, and each tube of cells was resuspended in 100 μL of dye (Alexa Fluor^®^ 647 Annexin V and Annexin Binding Buffer at a 1:100 ratio) and incubated at room temperature (25 ± 1 °C) for 20 min. Unbound dyes were removed by repeating centrifugation under identical conditions (600 × *g*, 5 min). Washed cells were resuspended in 500 μL of Annexin Binding Buffer, and 1 μL of Propidium Iodide (PI) was added to each tube and mixed thoroughly. After 5 min, cells in the early apoptotic phase (Annexin V^+^/PI^−^), late apoptotic phase (Annexin V^+^/PI^+^), and total apoptosis (the sum of early and late apoptosis) were analyzed using a flow cytometer, and the data were processed with FlowJo 10.6 software.

### Ki67 mean fluorescence intensity

Fluorescence-Activated Cell Sorting (FACS) was used to measure the mean fluorescence intensity of Ki67 in cells. Cells at a density of 1 × 10^5^ per well were seeded into 12-well plates and cultured until attachment. Following different treatments, the culture medium was discarded, and the cells were washed twice with PBS, followed by centrifugation at 600 × *g* for 5 min. The cells were fixed with 70% ethanol at −20 °C for 1 h, then centrifuged at 600 × *g* for 5 min. Cells were treated with 300 μL of membrane permeabilization buffer for 10 min, followed by centrifugation at 600 × *g* for 5 min. The cells were then resuspended in antibody dilution buffer (Ki67 diluted 1:100 in PBS) and incubated at room temperature for 30 min. After centrifugation at 600 × *g* for 5 min, the cells were incubated with a fluorescent secondary antibody (Alexa Fluor 488-conjugated goat anti-rabbit IgG, diluted 1:2,500 in PBS) for 1 h. The cells were washed twice with PBS, resuspended in 500 μL PBS, and analyzed by flow cytometry to detect Ki67-positive cells. Data were analyzed using FlowJo 10.6 software.

### Percentage of Lgr5^+^ cells

FACS was used to measure the number of Lgr5^+^ cells. IPEC-J2 cells were seeded into 12-well cell culture plates at a density of 1 × 10^5^ cells/well, with 1 mL of cell suspension per well. Following different treatments, cells were gently pipetted and collected into a centrifuge tube. The cells were centrifuged at 600 × *g* for 5 min, and the culture medium was discarded. The cells were resuspended in approximately 1 mL of PBS and centrifuged again at 600 × *g* for 5 min, discarding the supernatant. Each cell sample was incubated with 100 μL of Alexa Fluor^®^ 647-Lgr5 primary antibody dilution (Lgr5 diluted 1:250 in PBS) and incubated at room temperature in the dark for 20 min. Afterward, 900 μL of PBS was added to resuspend the cells, followed by centrifugation at 600 × *g* for 5 min and removal of the supernatant. The cells were resuspended in 500 μL PBS solution and analyzed by flow cytometry to determine the number of Lgr5^+^ stem cells. Data were analyzed using FlowJo 10.6 software.

### Cell cycle analysis

FACS was used to measure the cell cycle. Following different treatments, cells were carefully pipetted and collected into a centrifuge tube. The cells were centrifuged at 600 × *g* for 5 min, and the culture medium was discarded. Approximately 1 mL of ice-cold PBS was added to resuspend the cells, followed by centrifugation at 600 × *g* for 5 min and removal of the supernatant. The cells were then resuspended in 1 mL of pre-cooled 70% ethanol, mixed gently by pipetting, and fixed overnight at −20 °C. Afterward, the cells were centrifuged at 1,000 × *g* for 5 min, and the supernatant was discarded. Approximately 1 mL of ice-cold PBS was added to resuspend the cells, and the cells were centrifuged at 1,000 × *g* for 5 min, with the supernatant discarded. Each tube of cell samples was added to 0.5 mL of propidium iodide (PI) staining solution (prepared according to the kit instructions), and the cells were gently and thoroughly resuspended. The cells were incubated in the dark at 37 °C for 30 min. The cell cycle was analyzed by flow cytometry, and data were analyzed using FlowJo 10.6 software.

### Statistical analysis

The data were initially processed using Microsoft Excel 2019. Statistical analysis was performed using IBM SPSS Statistics 25. For in vivo experiments, data were analyzed using Student's *t*-test to compare differences between the control and DSS groups, as well as between the DSS and DSS + lactate groups. For the in vitro experiment, significant differences among multiple treatment groups were assessed using one-way analysis of variance (ANOVA) followed by Duncan’s multiple range test, while comparisons between two groups were conducted using Student's *t*-test. Detailed analysis methods are provided in the figure legends. Graphs were generated using GraphPad Prism 8.0. Data are presented as mean ± standard error of the mean (SEM). A statistically significant difference was considered at *P* < 0.05, a highly significant difference at *P* < 0.01, and an extremely significant difference at *P* < 0.001.

## Results

### Lactate alleviated jejunal structural damage and promoted intestinal proliferation in DSS-challenged piglets

**Fig. 1:**
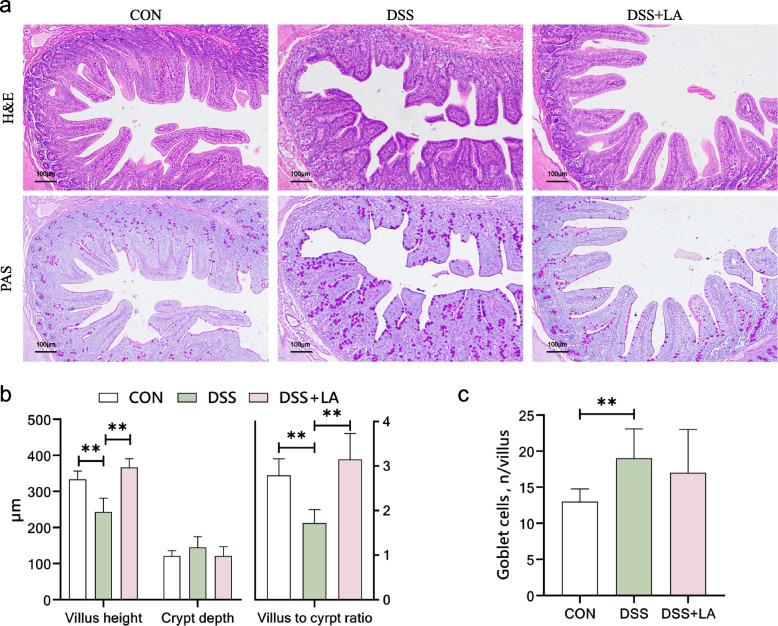
Effects of dietary supplementation with lactate (LA) on jejunal morphology in dextran sulfate sodium (DSS)-gavaging piglets. **a** Representative cross-sectional staining of the jejunum with haematoxylin and eosin (H&E) and periodic acid-Schiff (PAS) (× 100 magnification; scale bar: 100 μm). **b** Quantifying villus height and crypt depth in the jejunum (*n* = 10). **c** Quantifying the number of goblet cells per villus in the jejunum (*n* = 10). Statistical significance was calculated using Student's *t*-test. Results are expressed as means ± SEM. ^*^P < 0.05; ^**^P < 0.01

Histological assessment (PAS and H&E staining) showed that severe jejunal atrophy in DSS-treated piglets (Fig. 1a) was characterized by a 27% reduction in villus height (*P* < 0.05 vs. control) and a 38% decrease in villus-to-crypt ratio (*P* < 0.05) (Fig. 1b). In addition, DSS increased the number of goblet cells in the jejunal villi by 46% (*P* < 0.05 vs. control) (Fig. 1c). Lactate supplementation effectively counteracted these effects, restoring villus height to 101% of control levels (*P* < 0.05 vs. DSS group) and normalizing the villus-to-crypt ratio in the jejunum of piglets (*P* < 0.05 vs. DSS group). Lactate supplementation reduced goblet cell numbers by 10% (Fig. 1c); however, this change did not reach statistical significance (*P* > 0.05).

### Lactate activates Wnt/β-catenin signaling to drive epithelial renewal

**Fig. 2:**
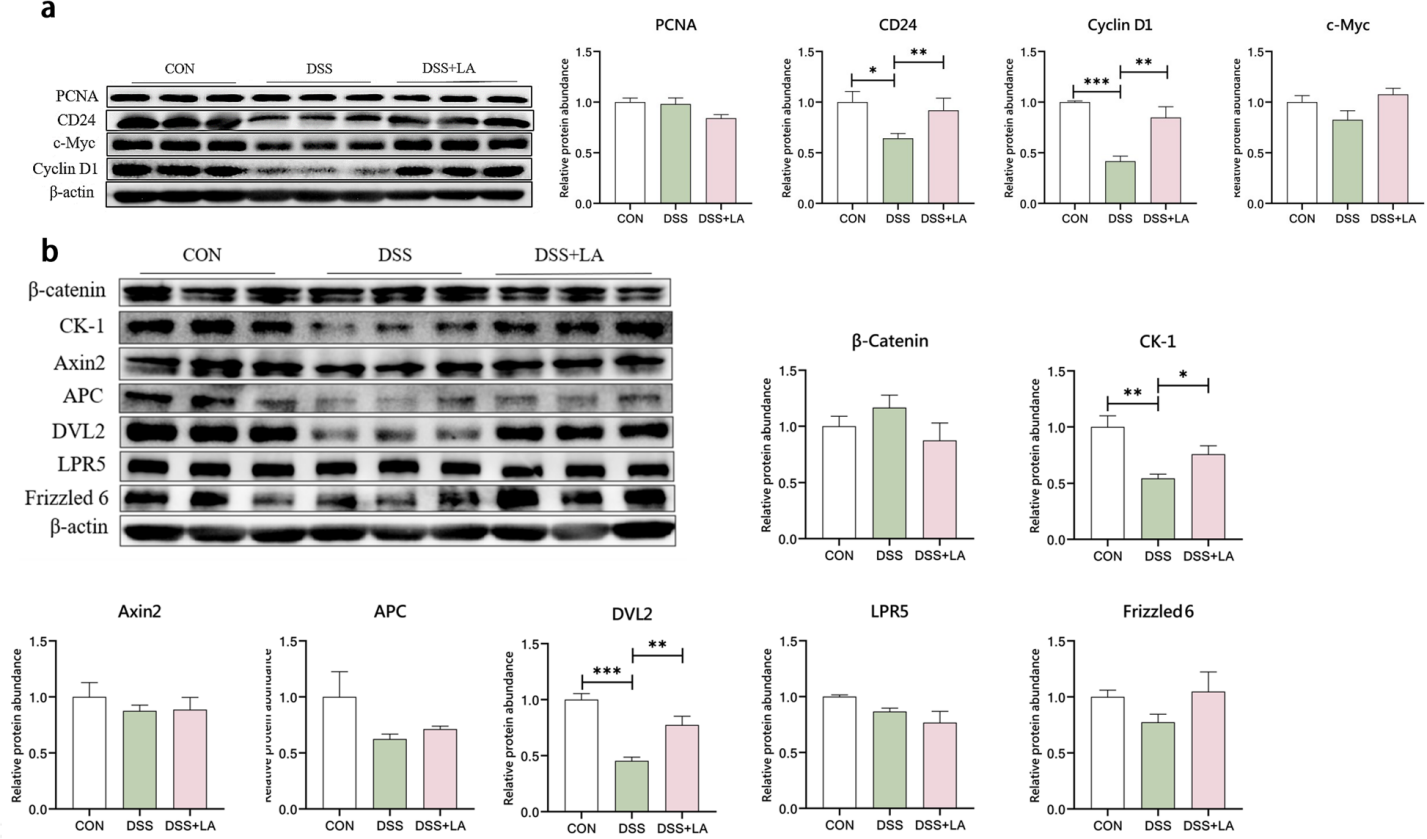
Effects of dietary supplementation with lactate (LA) on cell proliferation-related factors and Wnt/β-catenin pathway-related factors in the jejunum of DSS-gavaging piglets.** a** Representative cell proliferation-related factors in the jejunum, including proliferating cell nuclear antigen (PCNA), cluster of differentiation 24 (CD24), Cyclin D1, and v-myc avian myelocytomatosis viral oncogene homolog (c-Myc), were detected using Western blot, with actin serving as a loading control. Band quantification was performed to demonstrate PCNA, CD24, c-Myc, and Cyclin D1 protein levels in the jejunum (*n* = 3). **b** Western blot was used to detect representative Wnt/β-catenin pathway-related factors, including β-catenin, casein kinase 1 (CK-1), axin regulator of wnt signaling 2 (Axin2), adenomatous polyposis coli (APC), disheveled segment polarity protein 2 (DVL2), low-density lipoprotein receptor-related protein 5 (LRP5), and Frizzle 6, with actin as a loading control. Band quantification demonstrated the protein levels of β-catenin, CK1, Axin2, APC, DVL2, LPR5, and Frizzled 6 (*n* = 3). Statistical significance was calculated using Student's *t*-test. Results are expressed as means ± SEM. ^*^P < 0.05; ^**^P < 0.01; ^***^P < 0.001

Western blot analysis demonstrated that DSS suppressed proliferative capacity in the jejunum, with CD24 and Cyclin D1 protein levels reduced by 35% and 58%, respectively (*P* < 0.05) (Fig. 2a). Lactate administration reversed this inhibition, elevating CD24 level by 42% (*P* < 0.05) and Cyclin D1 level by 103% (*P* < 0.05) compared to DSS-treated counterparts. Western blot analysis revealed that DSS suppressed two critical Wnt pathway mediators in the jejunum (Fig. 2b), with CK-1 and DVL2 protein levels reduced by 46% and 55%, respectively (*P* < 0.05). Lactate administration reversed these deficits, elevating CK-1 level by 40% (*P* < 0.05) and DVL2 abundance by 70% (*P* < 0.05) compared to DSS-treated counterparts.

### Sodium lactate rescues DSS-impaired epithelial viability and attenuates DSS-induced pro-inflammatory cytokine production in vitro

**Fig. 3:**
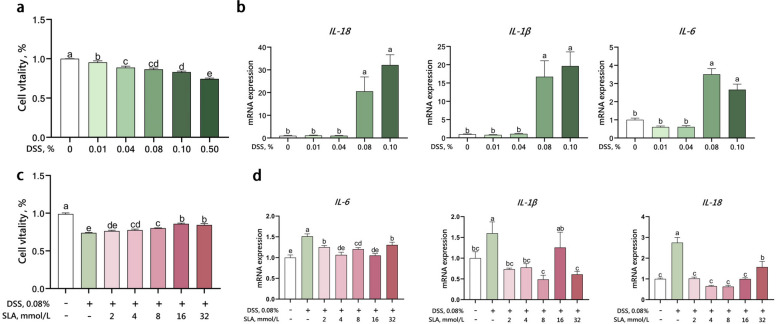
Sodium lactate attenuated gene expression of pro-inflammatory cytokines and restored cell viability in DSS-treated IPEC-J2 cells. Cells were treated with 0.01%, 0.04%, 0.08%, 0.1%, and 0.5% DSS for 24 h (*n* = 4). Cell viability was assessed using the CCK-8 assay (**a**), the mRNA expression levels of *IL-6*, *IL-1β*, and *IL-18* in 0.01%−0.1% DSS treated cells were quantified using RT-qPCR (**b**). Cells were treated with 0, 2, 4, 8, 16, and 32 mmol/L sodium lactate for 12 h. Subsequently, the cells were challenged with or without 0.08% DSS for 24 h (*n* = 4). After 24 h of challenge, cell viability was assessed using the CCK-8 assay (**c**), the mRNA expression levels of *IL-6*, *IL-1β*, and *IL-18* were quantified using RT-qPCR (**d**). Statistical significance was calculated via one-way analysis of variance (ANOVA) followed by Duncan’s multiple range test. Results are expressed as means ± SEM. ^a–e^Different letters on the bar charts indicate significant differences (*P* < 0.05)

In vitro, we first determined the effects of different DSS concentrations on IPEC-J2 cell viability and cytokine expression to establish an inflammatory model mimicking intestinal barrier dysfunction. The results showed that DSS dose-dependently compromises epithelial viability. Exposure to 0.01%–0.50% DSS for 24 h decreased the viability of IPEC-J2 cells by 5%–26% (*P* < 0.05 vs. untreated control) (Fig. 3a), and morphological observations under an optical microscope revealed that DSS visibly induced cell shrinkage (Fig. S1). RT-qPCR results (Fig. 3b) confirmed that 0.08% DSS was sufficient to significantly induce the overexpression of *IL-6*, *IL-1β*, and *IL-18* in IPEC-J2 cells (*P* < 0.05). Therefore, 0.08% DSS was selected for subsequent studies to establish an inflammatory model of intestinal epithelial cells.

Next, we evaluated the effect of lactate on cell viability. Lactate at concentrations of 2–16 mmol/L tended to enhance IPEC-J2 cell viability with or without DSS, although the results were not statistically significant (Fig. S2a). Subsequently, we treated IPEC-J2 cells with sodium lactate and hydrochloric acid to exclude the potential effect of the acidic environment caused by lactate on cell viability. Sodium lactate significantly enhanced IPEC-J2 cell viability, whereas hydrochloric acid markedly inhibited it (Fig. S2b). Based on these findings, we selected sodium lactate for co-treatment with DSS in IPEC-J2 cells.

Subsequently, we investigated the effects of co-treatment with sodium lactate and DSS on IPEC-J2 cell viability and cytokine expression. The results showed that the viability of IPEC-J2 cells in DSS-treated dropped to 73% of control (*P* < 0.05), whereas co-treatment with 4/8/16/32 mmol/L sodium lactate restored viability to 77%/80%/86%/85% (*P* < 0.01 vs. DSS alone) (Fig. 3d). Sodium lactate at concentrations of 2–32 mmol/L significantly attenuated (*P* < 0.05) DSS-induced cytokine expression of *IL-6*, *IL-1β*, and *IL-18* in IPEC-J2 cells (Fig. 3e), except the effect of 16 mmol/L sodium lactate on *IL-1β* expression was not significant.

### Sodium lactate restores DSS-impaired proliferation via stem cell activation and cell cycle progression in vitro

**Fig. 4:**
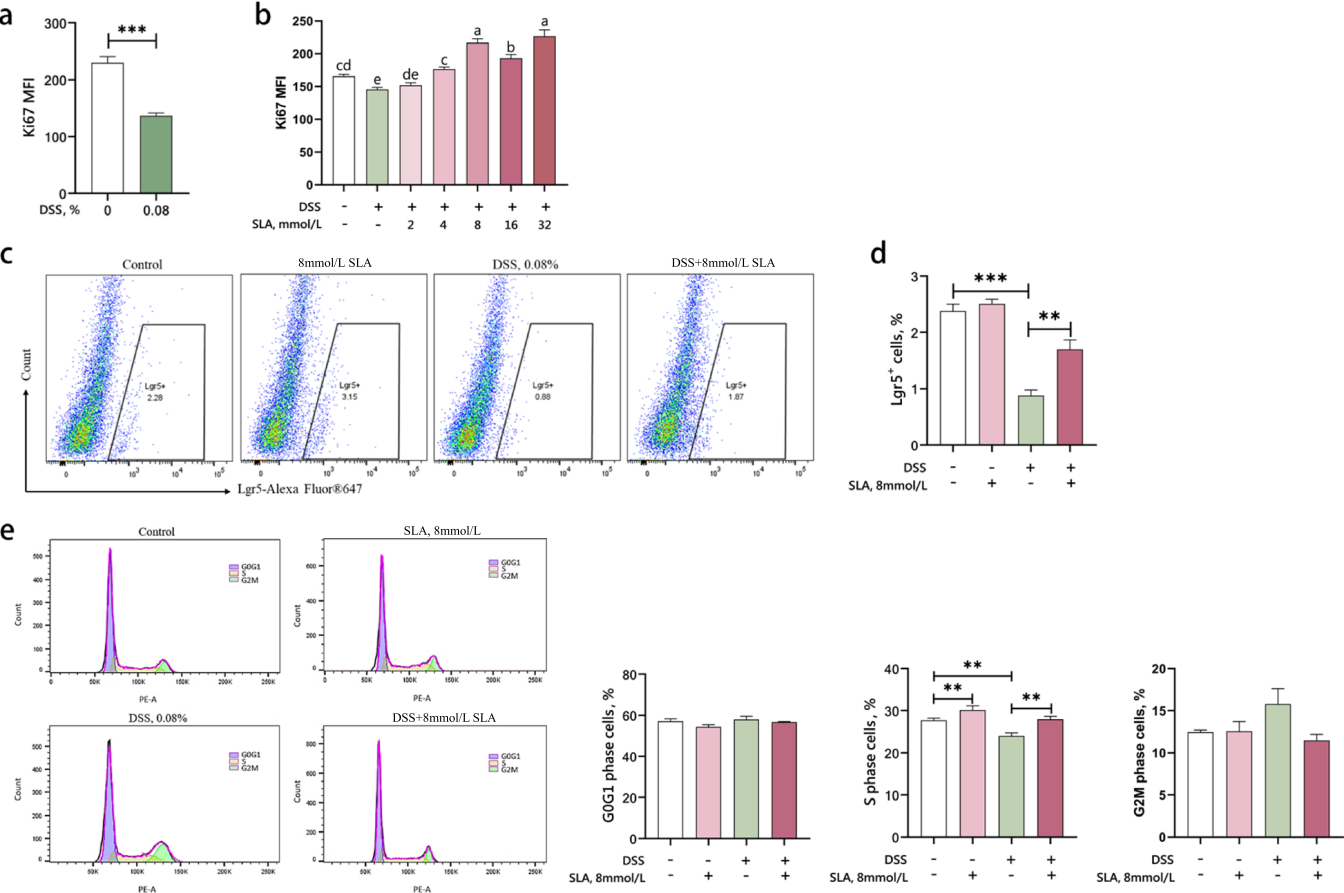
Sodium lactate rescued cell proliferation in DSS-treated IPEC-J2 cells. Cells were treated with 0.08% DSS for 24 h (*n* = 4). The mean fluorescence intensity (MFI) of Ki67 in IPEC-J2 cells was measured using flow cytometry (**a**). IPEC-J2 cells were treated with 0, 2, 4, 8, 16, and 32 mmol/L sodium lactate for 12 h. Subsequently, the cells were challenged with or without 0.08% DSS for 24 h (*n* = 4). After 24 h of challenge, the MFI of Ki67 in IPEC-J2 cells was measured using flow cytometry (**b**). Statistical significance was calculated using one-way analysis of variance (ANOVA) followed by Duncan’s multiple range test. ^a–e^Different letters on the bar charts indicate significant differences (*P* < 0.05; **b**). IPEC-J2 cells were treated with or without 8 mmol/L sodium lactate for 12 h, followed by treatment with or without 0.08% DSS for 24 h (*n* = 4). The proportion of Lgr5⁺ cells (**c **and** d**) and the cell cycle (**e**) were assessed using flow cytometry. Statistical significance was calculated using Student's *t*-test (**a**, **d** and **e**). Results are expressed as mean ± SEM. ^*^P <0.05; ^**^P <0.01; ^***^P < 0.001

Flow cytometry revealed that DSS suppressed the mean fluorescence intensity of Ki67 in IPEC-J2 cells (Fig. 4a). Sodium lactate (4–32 mmol/L) reversed this effect, increasing the mean fluorescence intensity of Ki67 by 50% at 8 mmol/L (*P* < 0.05) in IPEC-J2 cells co-treated with DSS (Fig. 4b).

Lgr5^+^ cells in DSS-treated IPEC-J2 cells dropped to 37% of control (*P* < 0.01), whereas co-treatment with 8 mmol/L sodium lactate restored Lgr5^+^ cells to 71% of control (*P* < 0.01 vs. DSS alone) (Fig. 4c and d). Cell cycle analysis demonstrated that sodium lactate (8 mmol/L) restored DSS-reduced S-phase cells (13% reduction by DSS) to 100% of control levels (*P* < 0.05) (Fig. 4e).

### Sodium lactate attenuates DSS-driven apoptosis in vitro

**Fig. 5:**
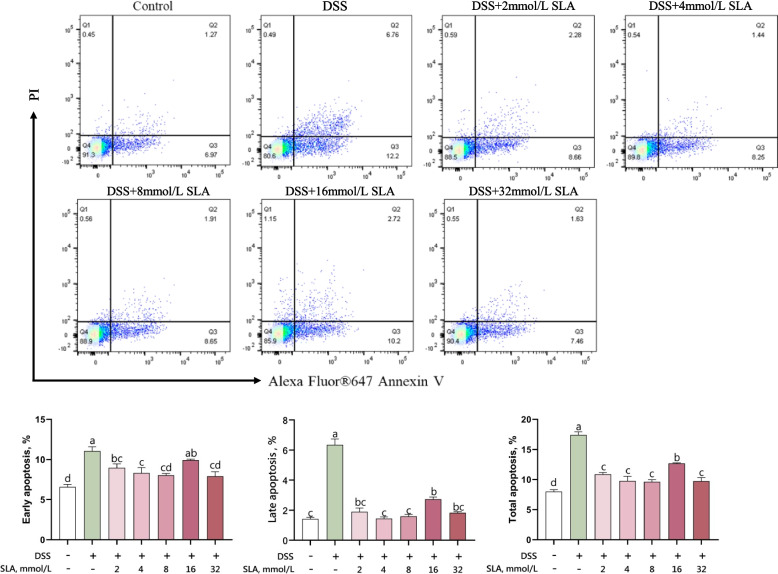
Sodium lactate attenuated cell apoptosis in DSS-treated IPEC-J2 cells. IPEC-J2 cells were treated with 0, 2, 4, 8, 16, and 32 mmol/L sodium lactate for 12 h. Subsequently, the cells were challenged with or without 0.08% DSS for 24 h (*n* = 4). After 24 h of challenge, cell apoptosis was assessed using flow cytometry. Statistical significance was calculated via one-way analysis of variance (ANOVA) followed by Duncan’s multiple range test. Results are expressed as means ± SEM. ^a–d^Different letters on the bar charts indicate significant differences (*P* < 0.05)

Building on the observed viability rescue (Fig. 3c), we dissected lactate's anti-apoptotic mechanism using Annexin V/PI flow cytometry (Fig. 5). DSS treated in IPEC-J2 cells for 24 h significantly increased (*P* < 0.05) early apoptosis, late apoptosis, and total apoptosis rates by 1.68, 4.44, and 2.17 times, respectively. Sodium lactate at concentrations of 2–32 mmol/L inhibited early apoptosis by 10%–28% (*P* < 0.05), late apoptosis by 57%–77% (*P* < 0.05), and total apoptosis by 27%–45% (*P* < 0.05) induced by DSS, except the effect of 16 mmol/L sodium lactate on early apoptosis was not significant.

## Discussion

The well-documented association between inflammatory bowel disease (IBD) and growth retardation underscores the clinical relevance of interventions that mitigate intestinal dysfunction [38]. Our previous study demonstrated that DSS-induced colitis in piglets significantly suppressed feed intake, impaired growth performance, and reduced feed conversion efficiency. Dietary lactate supplementation counteracted these adverse effects, rescuing growth performance [34]. The jejunum plays a central role in nutrient absorption, with villus height representing a key determinant of absorptive efficiency [39]. Structural disruption of the villi directly compromises nutrient uptake and contributes to growth retardation [40, 41]. Thus, the beneficial effects of lactate on growth performance during inflammation are likely mediated not only through modulation of the immune response [34, 42–44] but also by safeguarding jejunal villi against inflammatory injury. In recent years, multiple studies using various inflammatory models have demonstrated that lactate preserves colonic morphology against inflammatory injury [30, 31]; however, it remains unclear whether lactate can protect jejunal villi from inflammatory injury. In the present study, histopathological analyses provided direct evidence that DSS treatment induced a pronounced increase in goblet cell numbers within the jejunal villi, reflecting heightened immune activation, which was accompanied by severe villus atrophy. Dietary lactate supplementation, although exerting minimal effects on goblet cell abundance, restored villus height to levels comparable with the control group. These findings demonstrate that lactate mitigates inflammation-driven villus injury in the jejunum, providing a mechanistic explanation for its ability to alleviate DSS-induced growth impairment in piglets [34].

During inflammation, the integrity of the intestinal structure, the dynamic regulation of cell proliferation and apoptosis, and tissue renewal are closely related. Considering the protective effect of lactate on jejunal morphology, we hypothesized that lactate might promote tissue repair of DSS-induced damage by enhancing cell proliferation. By detecting key proteins associated with cell proliferation, we confirmed that DSS significantly reduced the protein levels of CD24 and Cyclin D1 in the jejunum of piglets. In contrast, lactate alleviated DSS-induced suppression of CD24 and Cyclin D1 expression. CD24, located upstream of Cyclin D1, can activate the Wnt/β-catenin pathway and influence Cyclin D1 expression, thereby promoting cell proliferation [45]. The consistent changes in CD24 and Cyclin D1 protein levels indicate that lactate may promote Wnt/β-catenin signaling. Subsequent experiments confirmed this hypothesis. DSS inhibited the expression of CK1 and DVL2, two key proteins in the Wnt pathway, whereas lactate mitigated DSS-induced suppression of CK1 and DVL2 expression. Notably, although the "destruction complex" involving CK1 and DVL2 mediates β-catenin phosphorylation and degradation through the proteasome [45], other studies have shown that CK1 and DVL phosphorylation are crucial for Wnt/β-catenin signaling in mammalian cells [46–48]. This phosphorylation enhances DVL signaling activity, thereby promoting Wnt/β-catenin signal transduction [46, 49, 50]. Additionally, research indicates that both overexpression and inhibition of CK1 can block Wnt signaling in mammals [49–51]. Therefore, the restoration of CK1 and DVL expression in the jejunum of DSS-treated piglets by lactate demonstrates that lactate-mediated Wnt/β-catenin signaling alleviates DSS-induced inflammatory damage in the jejunum. Previous studies have demonstrated that lactate can activate the Wnt/β-catenin signaling pathway in murine intestinal Paneth cells and stromal cells [33], and that activation of Wnt/β-catenin pathway contributes to the alleviation of inflammatory bowel disease in mammals [52]. Our study aligns with these findings and further confirms the role of the lactate-Wnt/β-catenin signaling pathway in alleviating inflammatory damage in the piglet intestine.

Previous studies have demonstrated that lactate can enhance GPR81 expression in porcine jejunal organoids, activate the Wnt/β-catenin signaling pathway, and promote ISCs proliferation [32]. However, it remains unclear whether lactate retains the capacity to restore impaired cell proliferation under inflammatory conditions. Our in vitro studies corroborated the in vivo findings, demonstrating lactate's capacity to ‌counteract DSS-induced epithelial dysfunction‌ through Wnt/β-Catenin-mediated mechanisms. On one side, the repair effects of lactate were further evidenced by its ability to ‌reverse the suppression of epithelial proliferation‌ caused by DSS. Ki67 immunostaining revealed that sodium lactate (4–32 mmol/L) ‌rescued DSS-impaired cell proliferation‌. These findings are consistent with the previous report [32] and further confirm that lactate can counteract the inhibitory effects of inflammation on intestinal epithelial cell proliferation. Under inflammatory conditions, Lgr5⁺ ISCs replenish damaged epithelial tissue through their rapid proliferation, thereby maintaining the structural integrity and barrier function of the intestine [16]. Patients with ulcerative colitis and mice with DSS-induced colitis show a reduction in Lgr5⁺ cells in the small intestine, impair epithelial self-renewal, and result in intestinal dysfunction [20, 21]. ‌ Previous study in a murine sepsis model has shown that induction of Lgr5⁺ expression within the intestine supports the preservation of colonic architecture [19], highlighting the role of intestinal Lgr5^+^ cells in maintaining epithelial integrity under inflammation. Mechanistically‌, Lgr5⁺ cells—critical Wnt/β-catenin effectors driving intestinal self-renewal [53, 54]—were depleted by DSS but ‌partially restored by sodium lactate co-treatment‌. ‌While sodium lactate alone did not expand Lgr5⁺ populations, its ‌synergistic interaction with Wnt signaling‌ under inflammatory stress likely stabilized β-catenin to reactivate Lgr5-dependent proliferative programs. This was further supported by cell cycle analysis. A complete cell cycle consists of DNA replication and cell division, including the G1 phase (before DNA synthesis), S phase (DNA synthesis), G2 phase (post-DNA synthesis), and M phase (cell division) [55]. The S phase represents a crucial cell cycle period in which chromosomal DNA is faithfully replicated, providing complete genetic information for the ensuing mitotic division (M phase) [56]. Our study demonstrated that lactate promotes the proliferation of IPEC-J2 cells by regulating the cell cycle and rescuing the DSS-induced depletion of Lgr5⁺ cells.

On the other hand, lactate-attenuating DSS-driven apoptosis‌ is another key contributor to preserving epithelial integrity‌. Excessive epithelial cell death is the driving factor in the pathogenesis of IBD, as the destruction of intestinal epithelial cells (IECs) compromises the intestinal barrier, increases the permeability of pathogenic factors, and triggers the release of damage-associated molecular patterns (DAMPs), exacerbating inflammatory injury [15, 57, 58]. Flow cytometry confirmed that DSS exposure significantly elevated early/late apoptosis rates, whereas sodium lactate co-treatment ‌reduced total apoptosis by 40%–60%‌. Previous studies have demonstrated that elevating the extracellular lactate-to-pyruvate ratio in tumor cells suppresses the JNK-Bax pathway by modulating the cytosolic NADH/NAD⁺ redox state, thereby preventing mitochondrial permeabilization and apoptosis [59]. Other reports have shown that the Wnt/β-catenin signaling pathway can inhibit caspase-dependent apoptosis [60, 61]. Consistent with these findings, the present study confirms the anti-apoptotic role of lactate and further provides evidence that lactate attenuates intestinal epithelial cell apoptosis via activation of the Wnt/β-catenin pathway. ‌Excessive IEC apoptosis also releases DAMPs that induce cytokine storm [15]—an effect confirmed by our in vitro experiments. Co-treatment with sodium lactate and DSS reduced the expression of *IL-6*, *IL-18*, and *IL-1β* in IPEC-J2 cells, indicating that the dual targeting of apoptosis and cytokine storm by lactate may synergistically slow the progression of IBD.

In addition, the maintenance of intestinal barrier homeostasis by lactate may be associated with cellular energy metabolism. Lactate has recently been identified as a substrate for the tricarboxylic acid (TCA) cycle [62], where it participates in mitochondrial energy metabolism. Lactate can also increase the NAD⁺/NADH ratio [63], thereby activating SIRT1 to promote the formation of mitochondrial complexes [63, 64], ultimately enhancing mitochondrial respiration. Published studies have demonstrated that enhanced mitochondrial oxidative phosphorylation increases ATP production and promotes the growth of IPEC-J2 cells [65]. In the present study, although lactate alone did not significantly improve cell viability, likely due to the acidic environment being unfavorable for IPEC-J2 cell growth [66], sodium lactate alone significantly enhanced cell viability. Under DSS-induced stress conditions, even a low dose of sodium lactate (4 mmol/L) was sufficient to restore cell viability, indicating increased lactate utilization by IPEC-J2 cells during inflammation. These results align with previous studies and further substantiate the role of lactate as a metabolic substrate in intestinal epithelial cells. Interestingly, previous studies have demonstrated a bidirectional interaction between mitochondrial energy metabolism and the Wnt/β-catenin signaling pathway, forming a “mitochondria–Wnt/β-catenin” regulatory network. For example, induction of the canonical Wnt pathway in megakaryocytes using LiCl (a GSK-3β inhibitor) was found to enhance mitochondrial mass in megakaryocytes [67]. Exogenous mitochondria were reported to activate Wnt signaling in hippocampal neural progenitor cells [68]. Conversely, inhibition of mitochondrial ATP production in zebrafish selectively suppressed Wnt/β-catenin signaling [69]. The mitochondria–Wnt/β-catenin network appears to maintain intestinal barrier homeostasis by regulating epithelial cell proliferation and apoptosis. Because activation of the Wnt/β-catenin pathway is closely associated with enhanced intestinal epithelial cell proliferation [33]. Moreover, highly proliferative ISCs possess more mitochondria than their neighboring cells [20, 62], and shifting cellular metabolism toward mitochondrial respiration enhances ISC self-renewal and differentiation, thereby mitigating disease-associated damage [20]. Conversely, mitochondrial dysfunction results in the depletion of Lgr5⁺ ISCs and exacerbates IBD progression [20], while inhibition of mitochondrial function increases abnormal apoptosis in intestinal epithelial cells. These studies provide compelling evidence for a “mitochondria–Wnt/β-catenin–cell proliferation/apoptosis” axis. Our findings integrate lactate, a key energy metabolite, into this network, further supporting the link between energy metabolism and the proliferation/apoptosis-driven processes that govern IBD progression and post-inflammatory tissue repair.

## Conclusion

In summary, this study demonstrates that lactate restores DSS-impaired Wnt/β-catenin signaling in the jejunum of piglets, thereby regulating the cell cycle, promoting Lgr5⁺ cell proliferation, and suppressing DSS-induced excessive apoptosis, ultimately alleviating morphological damage to the jejunal epithelium. Our findings confirm the critical role of lactate in protecting the intestinal barrier through the regulation of epithelial cell proliferation and apoptosis, and elucidate a mechanism by which lactate rescues growth performance in piglets with intestinal inflammation. These results provide novel theoretical support for the potential application of lactate in managing inflammatory bowel disease in piglets. Nevertheless, whether the protective effects of lactate are also mediated by enhancing mitochondrial function to promote cell proliferation and apoptosis regulation warrants further investigation.

## Supplementary Information

Additional file 1: Fig. S1. DSS induced cell shrinkage in IPEC-J2 cells. Cells were treated with 0.01%, 0.04%, 0.08%, 0.1%, and 0.5% DSS for 24 h (*n* = 4). IPEC-J2 cell morphology was observed using an inverted fluorescence microscope (Olympus CKX53, Olympus Corporation, Tokyo, Japan). Fig. S2. (a) IPEC-J2 cells were treated with 0, 2, 4, 8, 16, and 32 mmol/L lactate for 12 h (*n* = 4). IPEC-J2 cells were challenged with or without 0.08% DSS for 24 h (*n* = 4). Cell viability was assessed using the CCK-8 assay. Statistical significance was calculated via one-way analysis of variance (ANOVA) followed by Duncan’s multiple range test. ^a–c^Different letters on the bar charts indicate significant differences (*P* < 0.05). Results are expressed as means ± SEM. (b) IPEC-J2 cells were treated with or without 8 mmol/L lactate or hydrochloric acid (HCL) for 12 h, followed by treatment with or without 0.08% DSS for 24 h (*n* = 4). Statistical significance was calculated using Student's *t*-test (c, d). Results are expressed as means ± SEM.

Additional file 2: Table S1. Ingredients and composition of basal diets. Table S2. Primer sequences of IPEC-J2 cells used for real-time PCR.

Additional file 3. Original images for Western blot.

## Data Availability

All data generated or analyzed during this study are available from the corresponding author upon reasonable request.

## Abbreviations

APC: Adenomatous polyposis coli
ATCC: American type culture collection
Bax: Bcl-2-associated X protein
CCK8: Cell Counting Kit-8
CD24: Cluster of differentiation 24
CK-1: Casein kinase 1
c-Myc: Cellular myelocytomatosis virus
DSS: Dextran sulfate sodium
DVL2: Dishevelled-2
FACS: Fluorescence-activated cell sorting
GSK3β: Glycogen synthase kinase-3β
H&E: Hematoxylin and eosin
IBD: Inflammatory bowel disease
IL: Interleukin
ISCs: Intestinal stem cells
I-FABP: Intestinal fatty acid-binding protein
JNK: C-jun n-terminal kinase
LA: Lactate
Lgr5: Leucine-rich repeat-containing G-protein coupled receptor 5
LPS: Lipopolysaccharide
LPR5: Low-density lipoprotein receptor-related protein 5
PCNA: Proliferating cell nuclear antigen
PVDF: Polyvinylidene fluoride
SIRT: Sirtuin
SLA: Sodium lactate
STAT: Signal transducer and activator of transcription
TCA: Tricarboxylic acid
TFAM: Mitochondrial transcription factor A
TNBS: 2,4,6-Trinitrobenzenesulfonic acid
Wnt: Wingless/Integrated
