# Supplementary figures and images for: Lactate alleviates intestinal barrier injury in weaned piglets via activation of the Wnt/β-catenin pathway and promotion of intestinal epithelial cell proliferation
Source: J Anim Sci Biotechnol. 2025 Nov 28;16:160. doi: 10.1186/s40104-025-01290-x (PMC12661717; doi:10.1186/s40104-025-01290-x)

Supplementary Figure 1

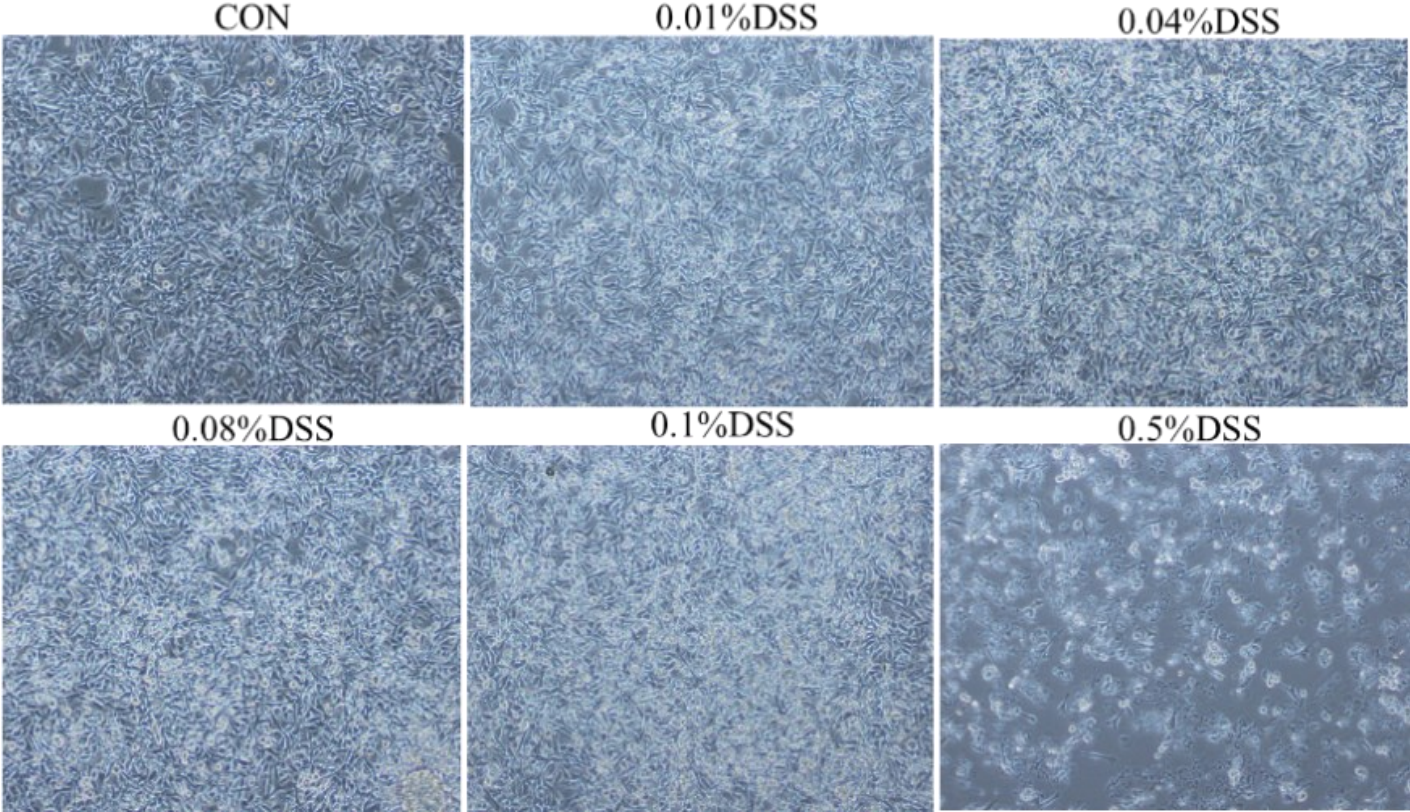

Supplementary Figure 2

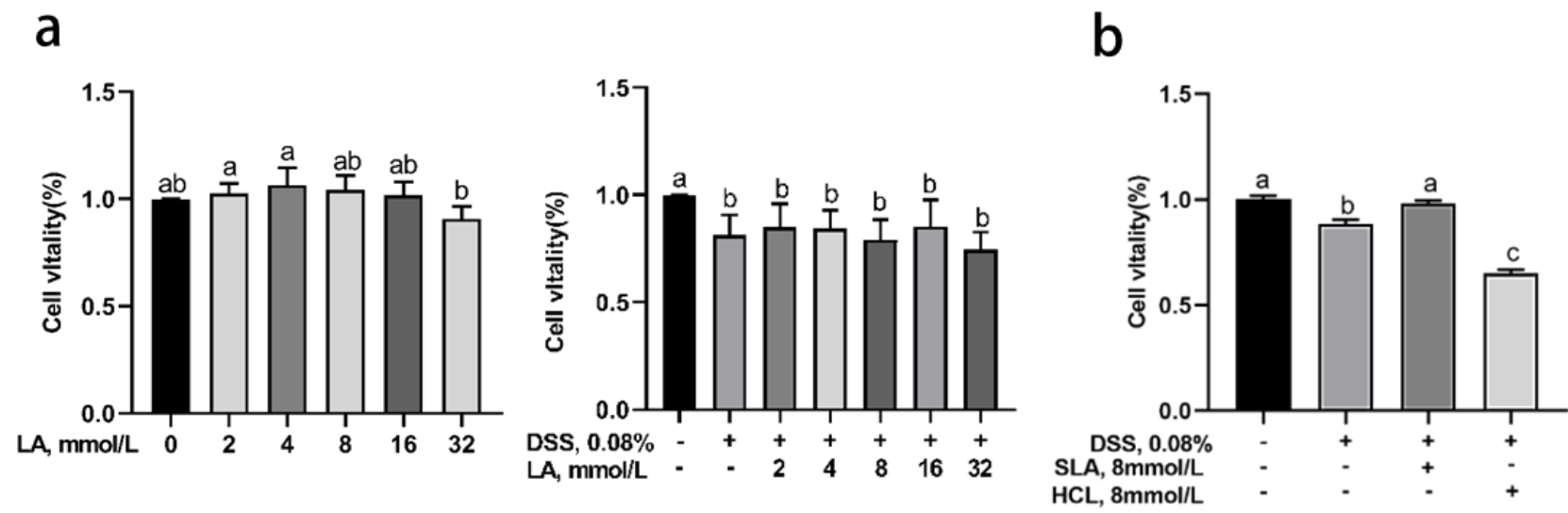

Supplement: Supplementary file 1 — Additional file 1: Fig. S1. DSS induced cell shrinkage in IPEC-J2 cells. Cells were treated with 0.01%, 0.04%, 0.08%, 0.1%, and 0.5% DSS for 24 h (n = 4). IPEC-J2 cell morphology was observed using an inverted fluorescence microscope (Olympus CKX53, Olympus Corporation, Tokyo, Japan). Fig. S2. (a) IPEC-J2 cells were treated with 0, 2, 4, 8, 16, and 32 mmol/L lactate for 12 h (n = 4). IPEC-J2 cells were challenged with or without 0.08% DSS for 24 h (n = 4). Cell viability was assessed using the CCK-8 assay. Statistical significance was calculated via one-way analysis of variance (ANOVA) followed by Duncan’s multiple range test. a–cDifferent letters on the bar charts indicate significant differences (P < 0.05). Results are expressed as means ± SEM. (b) IPEC-J2 cells were treated with or without 8 mmol/L lactate or hydrochloric acid (HCL) for 12 h, followed by treatment with or without 0.08% DSS for 24 h (n = 4). Statistical significance was calculated using Student's t-test (c, d). Results are expressed as means ± SEM. [file 40104_2025_1290_MOESM1_ESM.pdf]
