# Supplementary material for: Lactate alleviates intestinal barrier injury in weaned piglets via activation of the Wnt/β-catenin pathway and promotion of intestinal epithelial cell proliferation
Source: J Anim Sci Biotechnol. 2025 Nov 28;16:160. doi: 10.1186/s40104-025-01290-x (PMC12661717; doi:10.1186/s40104-025-01290-x)
Supplement: Supplementary file 3 — Additional file 3. Original images for Western blot. [file 40104_2025_1290_MOESM3_ESM.pdf]

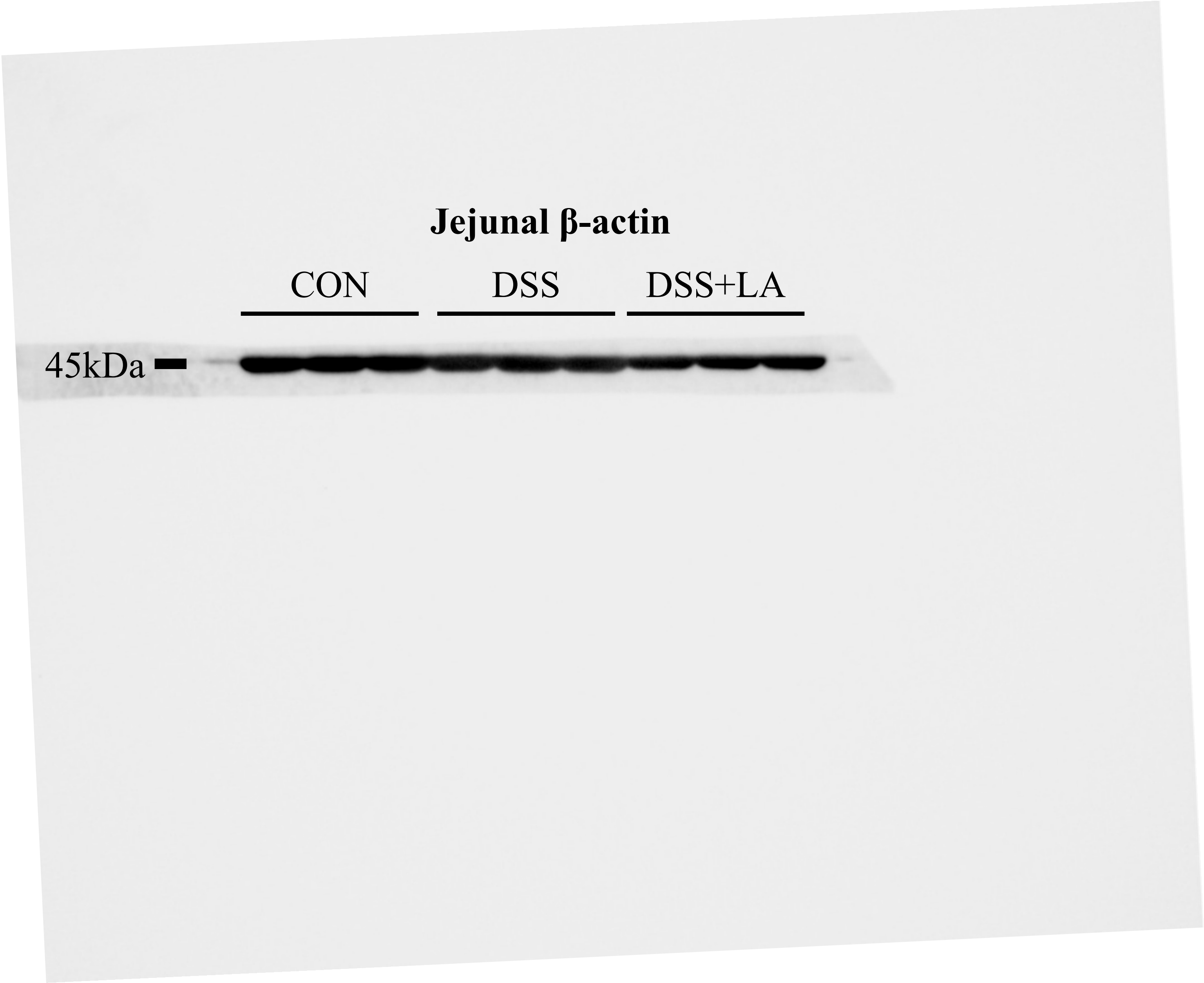
Jejunal β-actin
CON
DSS
DSS+LA
45kDa

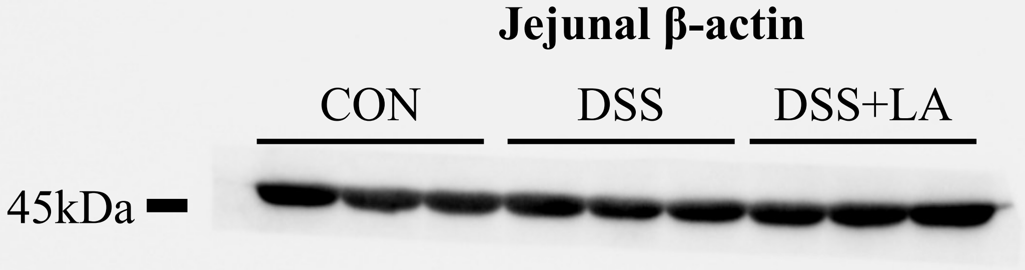
Jejunal β-actin
CON
DSS
DSS+LA
45kDa

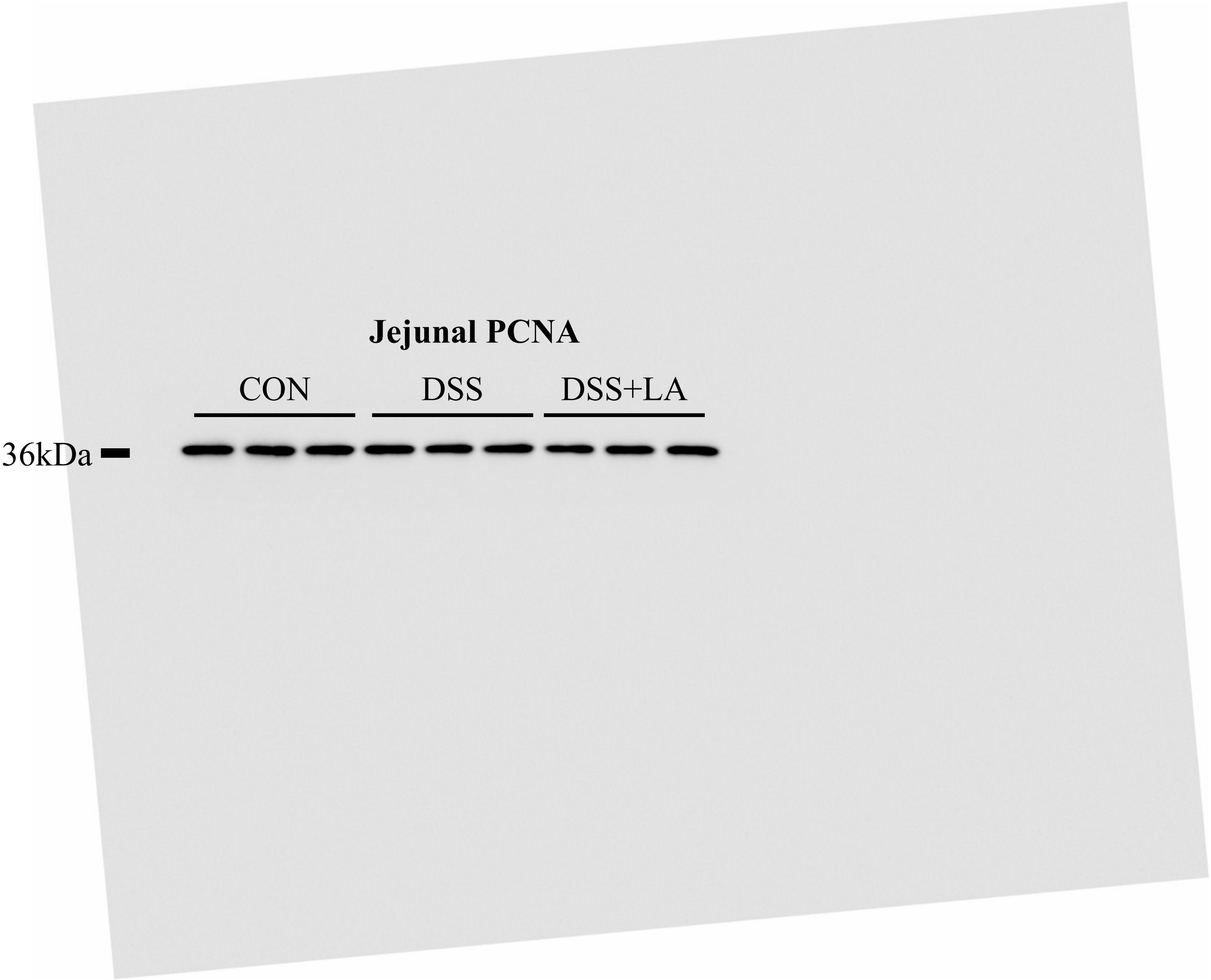
Jejunal PCNA
CON
DSS
DSS+LA
36kDa

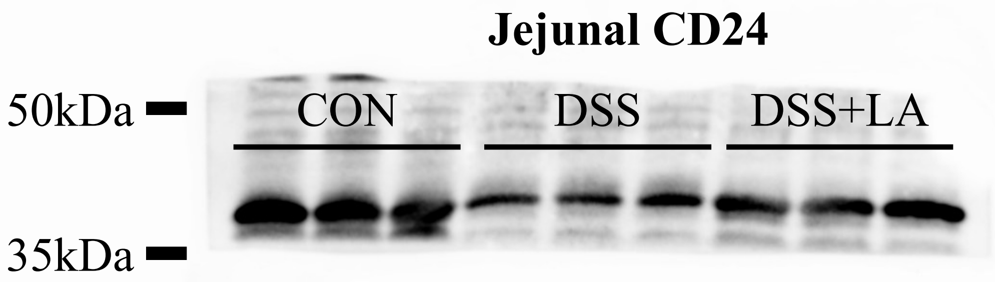
Jejunal CD24
50kDa
35kDa
CON
DSS
DSS+LA

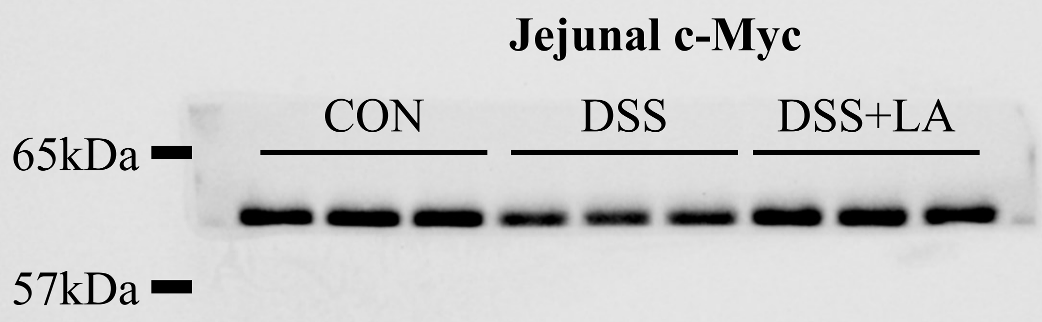
Jejunal c-Myc
CON
DSS
DSS+LA
65kDa
57kDa

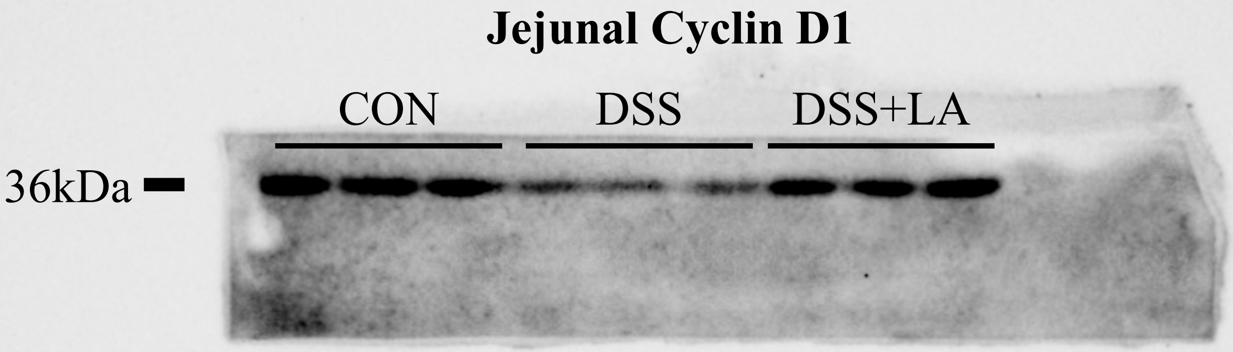
Jejunal Cyclin D1
CON
DSS
DSS+LA
36kDa

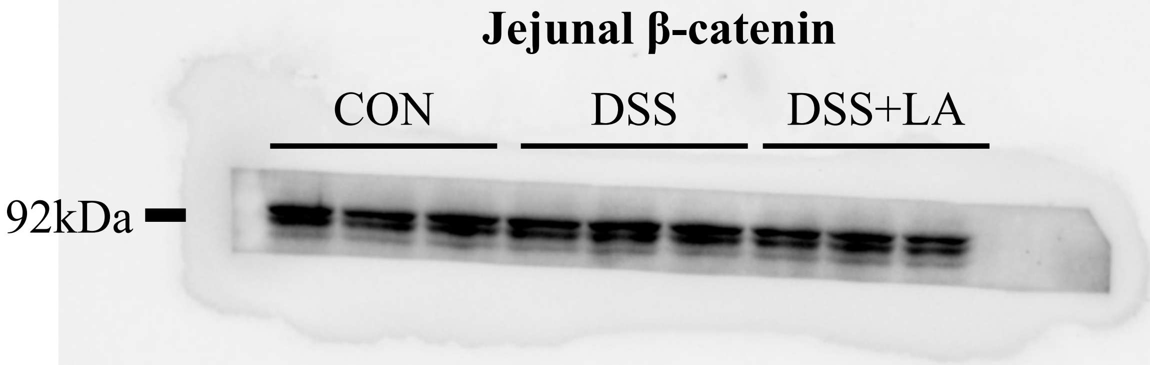

Jejunal β-catenin
CON
DSS
DSS+LA
92kDa

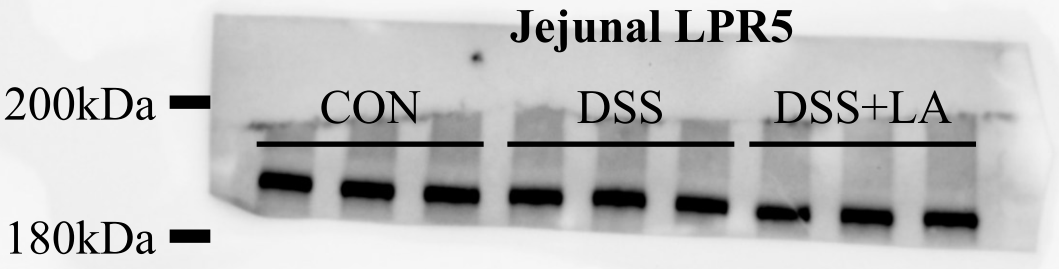
Jejunal LPR5
200kDa
180kDa
CON
DSS
DSS+LA

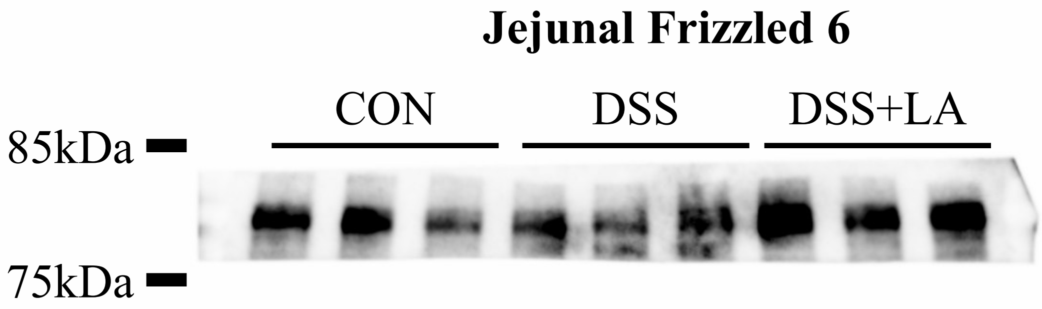
Jejunal Frizzled 6
CON
DSS
DSS+LA
85kDa
75kDa

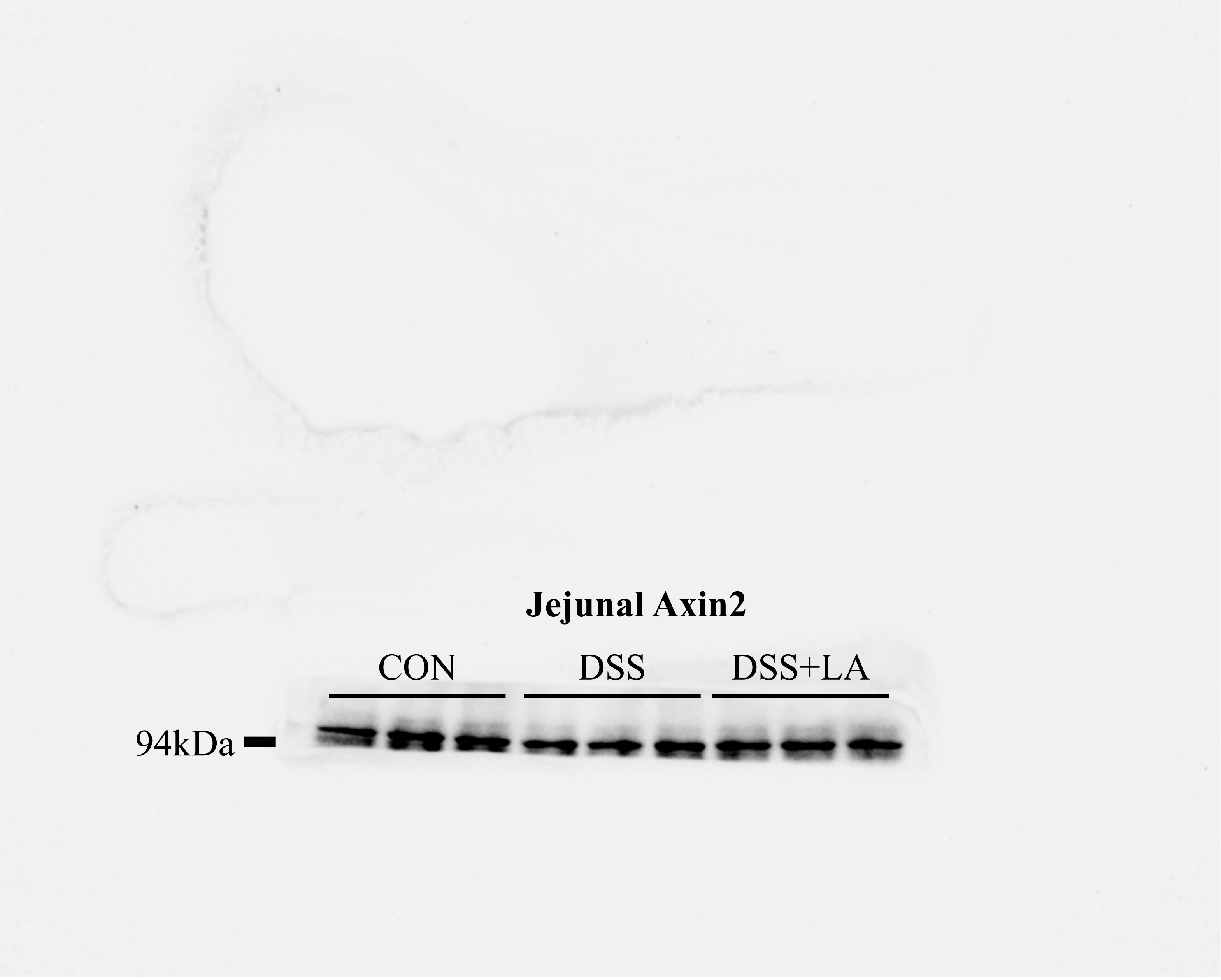
Jejunal Axin2
CON
DSS
DSS+LA
94kDa

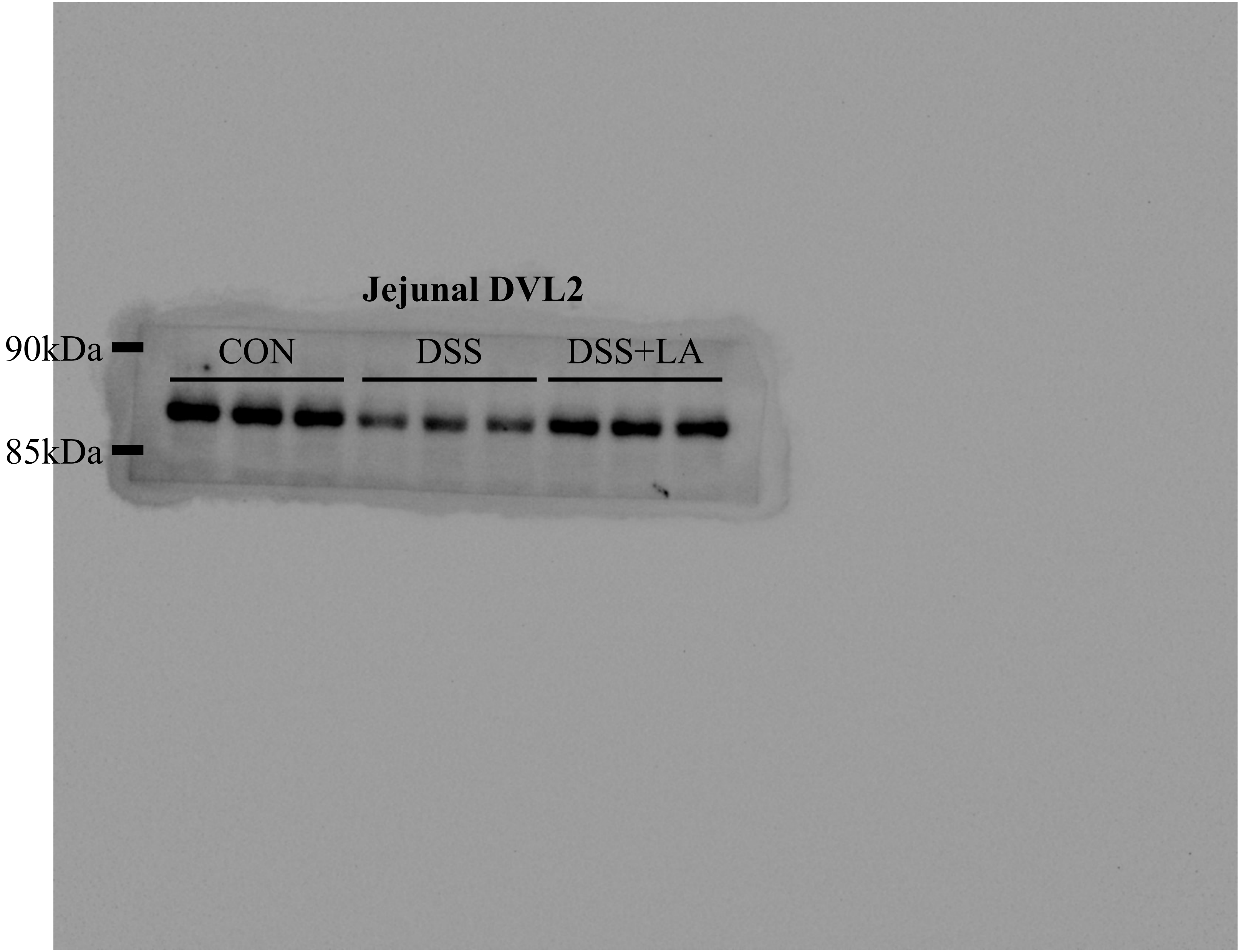
Jejunal DVL2
90kDa
85kDa
CON
DSS
DSS+LA

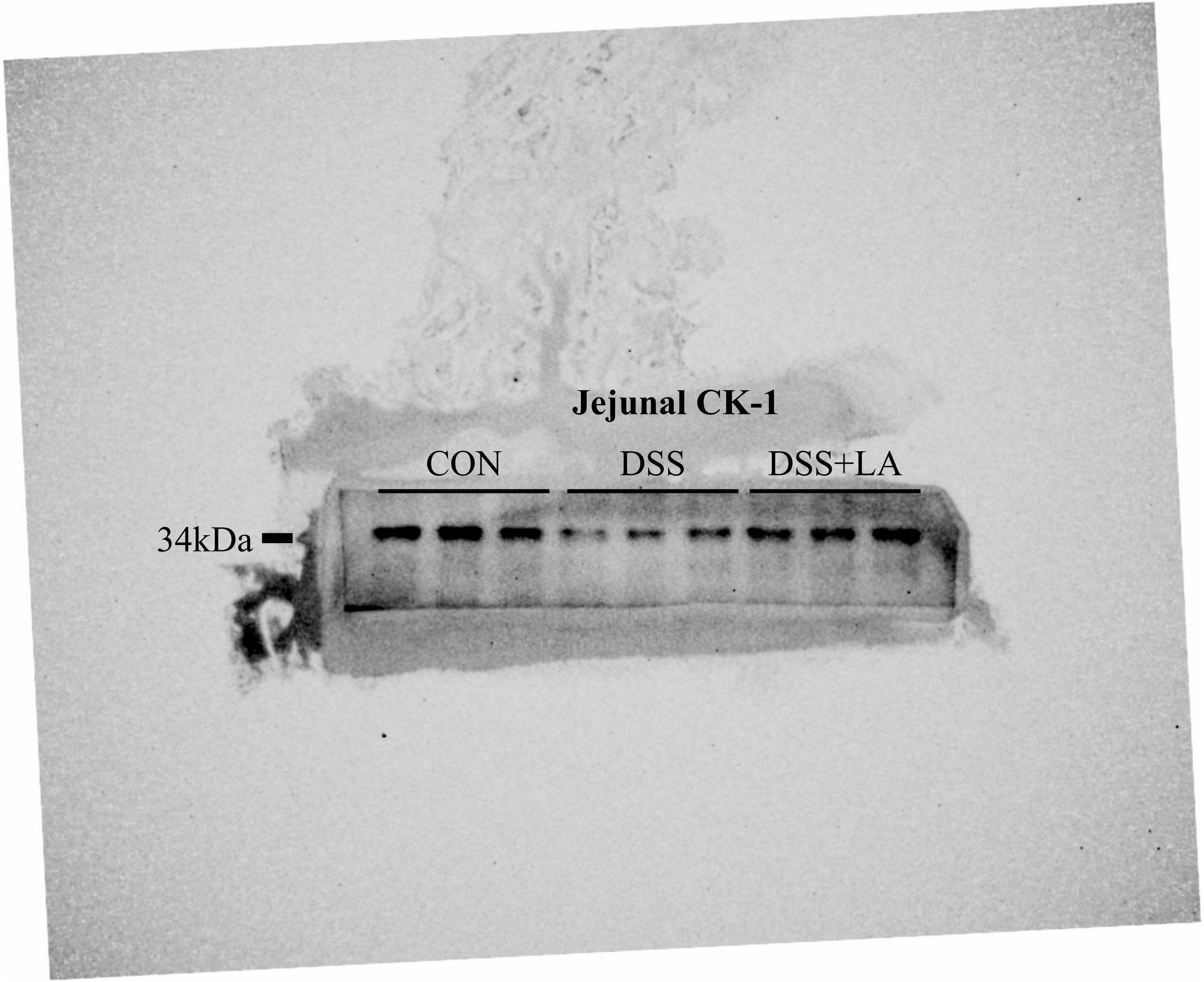

Jejunal CK-1
CON
DSS
DSS+LA
34kDa

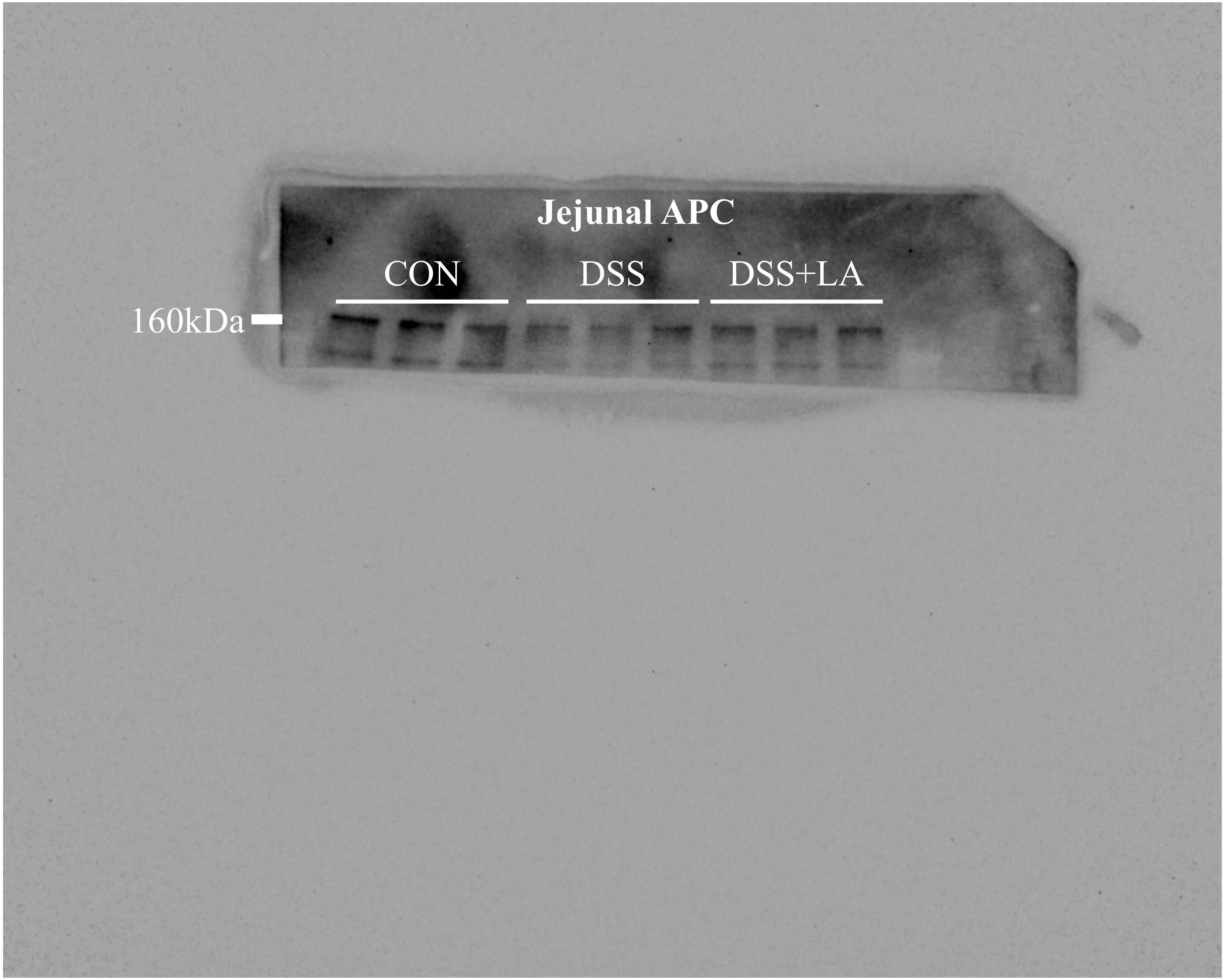
Jejunal APC
CON
DSS
DSS+LA
160kDa
